# P-1776. Impact of Sepsis Recognition Tools and Pharmacy Workflow on Sepsis Management and Outcomes

**DOI:** 10.1093/ofid/ofae631.1939

**Published:** 2025-01-29

**Authors:** Salma Abdelmahab, Yanina Dubrovskaya, Kassandra Marsh, Cristian Merchan, Justin Siegfried, John Papadopoulos, Samantha Smalley

**Affiliations:** NYU Langone Health, New York, New York; NYU Langone Health, New York, New York; NYU Langone Health, New York, New York; NYU Langone Health, New York, New York; NYU Langone Health, New York, New York; NYU Langone Health, New York, New York; NYULH, New York, New York

## Abstract

**Background:**

CDC 2023 Hospital Sepsis Program Core Elements prioritizes protocolized care, timely and effective antibiotics and de-escalation. At NYULH, robust sepsis tools integrated into Epic since July 2021 include the Sepsis Indicator Column on the Emergency Department (ED) Track Board for patient identification, nurse and physician-driven Best Practice Advisory (BPA), ED Sepsis Quick List for antimicrobial orders, and Sepsis Guidelines. Sepsis antibiotics in the ED are administered as IV push over 3 minutes followed by IV piggyback since March 2021.

**Figure:**
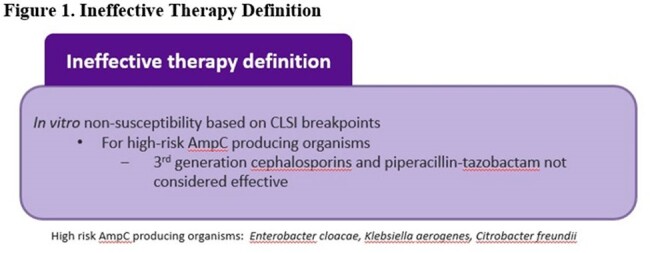

**Methods:**

We conducted a retrospective review of adult patients with community-onset sepsis and confirmed bacteremia who presented to ED between 9/2022 and 5/2023. The primary outcomes were time to antibiotics and the percent of patients with ineffective empiric antibiotics. Secondary outcomes included time to de-escalation and sepsis outcomes. As a process measure, we evaluated sepsis guidelines adherence and pharmacy interventions.

**Figure:**
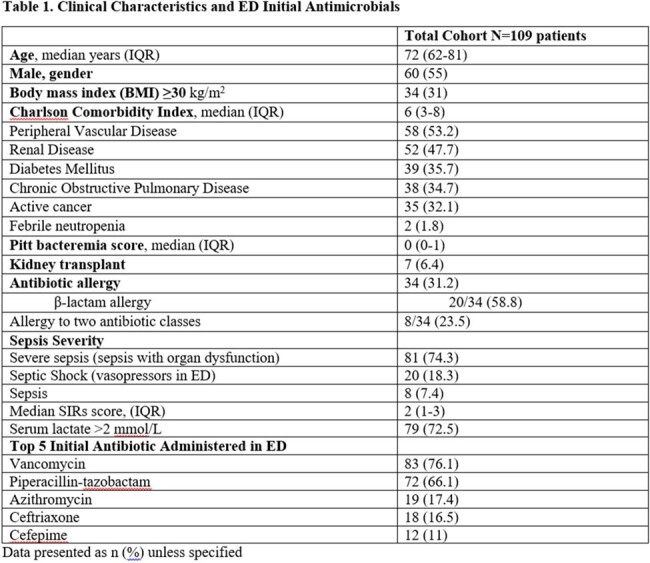

**Results:**

During 9/2022 to 8/2023, 1682 ED sepsis cases were recorded, and 22% had bacteremia. Among 109 included patients, median Charlson Comorbidity Index was 6 (IQR 3-8) and Pitt bacteremia score 0 (IQR 0-1). Median time from ED sepsis recognition to antibiotic verification by pharmacy was 9 min (IQR 0-27) with median time to antibiotic administration of 29 min (IQR 15-50). Ineffective initial therapy occurred in 11% of cases with median time to effective antibiotics at 24 h. De-escalation was seen in 84% of cases with median time of 2 days for both MRSA and atypical coverage discontinuation and 4 days for *Pseudomonas* coverage. Initial therapy adhered to guidelines in 70.6% of cases, and deviation primarily occurred due to the administration of vancomycin in the absence of MRSA risk factors. Antibiotic-related pharmacy and stewardship (ASP) recommendations were made in 40% of patients while in ED and 96% of patients during hospital stay. In-hospital mortality, ICU admission, 30-day infection related re-admission and *C. difficile* infection within 6 months were 8.3%, 42.2%, 8.3% and 1.8%, respectively.

**Figure:**
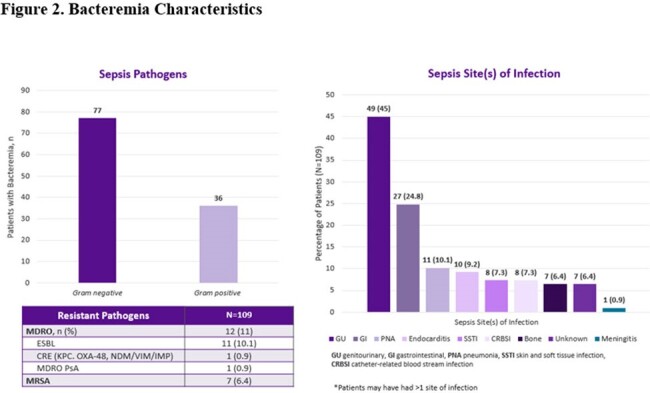

**Conclusion:**

The integration of Epic sepsis recognition tools with ASP and pharmacy workflows led to high rates of timely and effective antibiotic administration in the ED and de-escalation during hospitalization.

**Figure:**
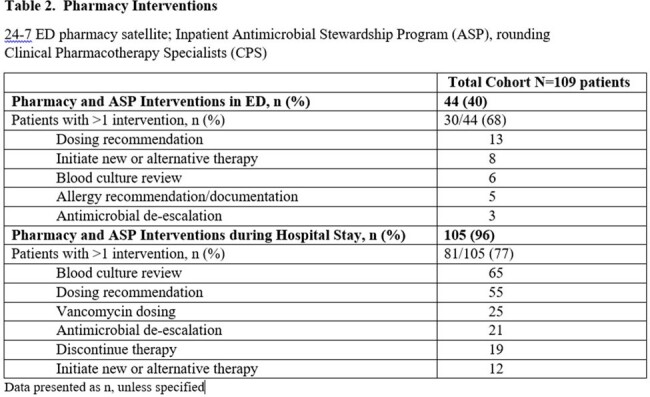

**Disclosures:**

**All Authors**: No reported disclosures

